# Successful Hiatal Hernia Repair in a 95-Year-Old Patient: A Case Report

**DOI:** 10.7759/cureus.69375

**Published:** 2024-09-13

**Authors:** Zofia Zielińska, Anna Ruszecka, Mateusz Kęska, Wojciech Czajkowski

**Affiliations:** 1 Thoracic Surgery, Jagiellonian University Medical College, Kraków, POL; 2 Medical and Health Sciences, Jagiellonian University Medical College, Kraków, POL; 3 Thoracic Surgery, Pulmonary Hospital "Odrodzenie", Zakopane, POL

**Keywords:** case report, elderly patient, incarcerated hiatal hernia, nissen fundoplication, surgical repair

## Abstract

A sliding hiatal hernia is a frequent pathology. Its prevalence increases with age. However, incarceration of such hernias is a very rare phenomenon with a lack of precise data in the literature. Should this condition occur, it requires immediate surgical intervention. We report a case of a 95-year-old female presenting with thoracic pain and general state deterioration. The patient was otherwise relatively stable on admission. Causes of cardiac origin were excluded. A plain chest X-ray and a chest computed tomography (CT) revealed a giant sliding incarcerated hiatal hernia. Urgent laparotomy was performed, and the hernia strangulation was confirmed. The rim of the esophageal hiatus was cut to enable the repositioning of the stomach. No necrosis was found. The hiatal crura was approximated with sutures, and the Nissen fundoplication was performed successfully with no postoperative complications. The patient was discharged in good condition. Surgical procedures in the elderly pose a great challenge due to numerous risk factors, such as polypharmacy, comorbidities, and frailty syndrome. However, an incarcerated hiatal hernia is a condition that necessitates urgent surgery, either through laparoscopy or laparotomy. It can be easily missed, mimicking cardiac symptoms. In this case, the ailment was successfully diagnosed, the laparotomy approach was chosen, and the Nissen fundoplication was performed to prevent possible gastroesophageal reflux disease (GERD).

## Introduction

There are four types of diaphragmatic hiatal hernias as follows: sliding, paraesophageal, mixed, and those accompanied by displacement of other organs. A sliding hiatal hernia appears when an upper part of the stomach moves upwards through the widened esophageal hiatus with a displacement of the gastroesophageal junction to the chest. Despite this type of hernia being quite common, its exact prevalence in the general population is yet to be established. According to a study from 2021, almost 10% of patients undergoing lung CT may have a hiatal hernia [1]. Dunn et al. reported their prevalence at 15-20% among the people of Western countries [2]. The predominant type is the sliding hernia (85-95%) [3]. While most hiatal hernias tend to be asymptomatic, they may also manifest as gastroesophageal reflux disease with signs such as heartburn, regurgitation, or in advanced cases, chest pain, vomiting, dyspnea, and dysphagia [3,4]. The most popular diagnostic tools for this ailment are endoscopy, radiographic studies, or manometry. However, it is quite often an incidental finding on CT [3,5]. Hiatal hernia incarceration is a very rare but life-threatening medical condition, with no exact data on its prevalence available in the literature [2]. Nevertheless, should such an event occur, the resulting necrosis may lead to organ rupture and would be perceived as a surgical emergency. We present a case of a 95-year-old female with a symptomatic, incarcerated, sliding hiatal hernia. The patient was successfully diagnosed and surgically treated. Nonetheless, the described example is very uncommon and could have easily been misdiagnosed and mistreated. Therefore, it is crucial to identify this life-threatening emergency as a differential diagnosis, which can oftentimes be managed surgically with great success.

## Case presentation

**Figure 1:**
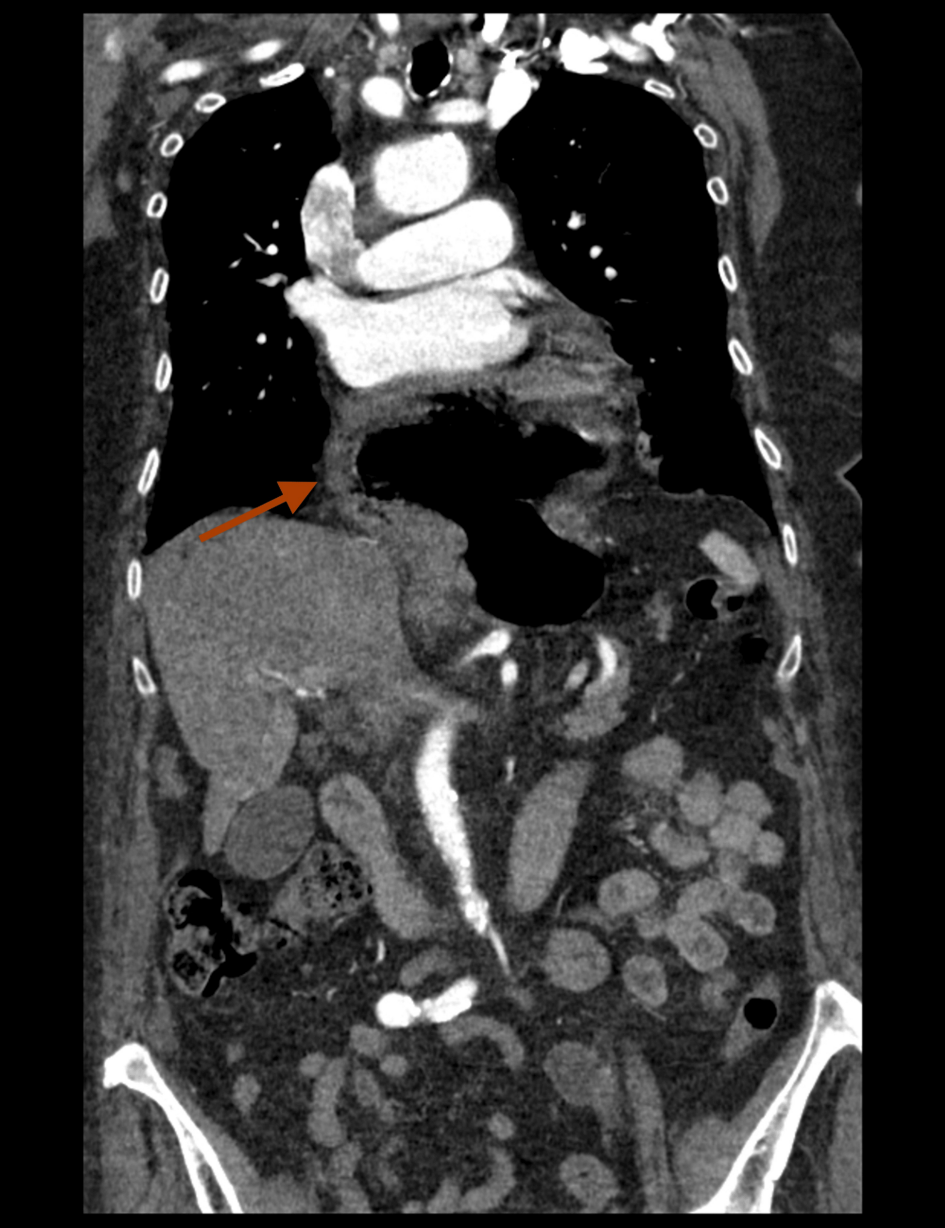
Chest CT showing incarcerated sliding hiatal hernia (coronal). The red arrow points to the displacement of the gastric fundus into the thoracic cavity above the diaphragm. The hernia compresses the heart and lower lung fields.

**Figure 2:**
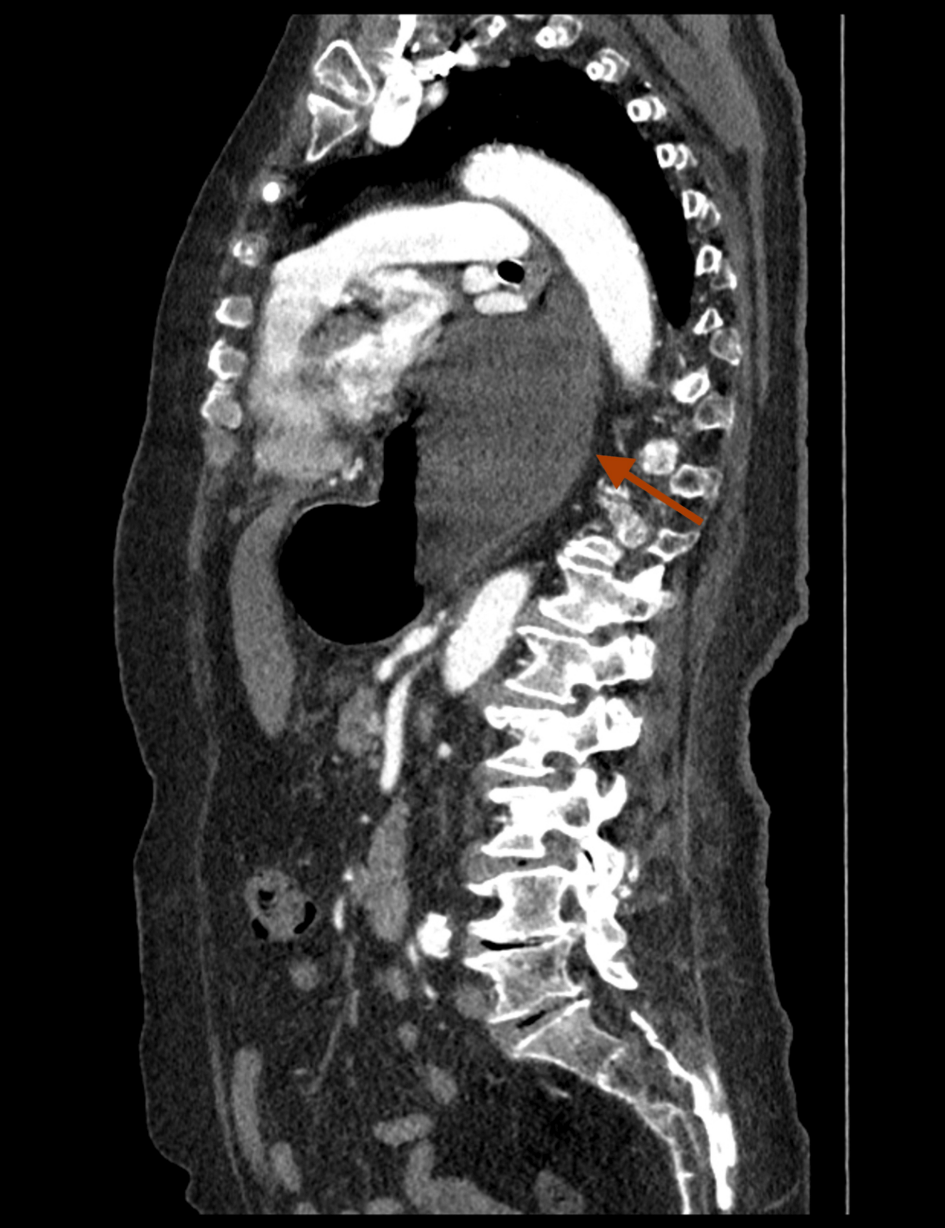
Chest CT showing incarcerated sliding hiatal hernia (sagittal). The red arrow indicates the hernia, visible as a retrocardiac mass. The presence of an air-fluid level within the mass supports the diagnosis.

A 95-year-old female patient was referred to the Department of Thoracic Surgery, the Pulmonary Hospital "Odrodzenie" in Zakopane, for an urgent incarcerated hiatal hernia repair. She presented to the emergency room with recent general health status deterioration, dyspnea, resting chest pain, and dysphagia. The patient’s past medical history was relevant for arterial hypertension and chronic obstructive pulmonary disease with a smoking history. Her medication comprised a combination of a beta-blocker, a diuretic, an angiotensin receptor blocker, and inhalations with a corticosteroid and a sympathomimetic. Further anamnesis was insignificant for any other important risk factors. On admission, the patient was alert and oriented, and her vital signs were stable. Her calculated body mass index amounted to 32.05 kg/m², which classified her as class I obese. Physical examination revealed mild tachypnea with no other relevant findings. Initial main differential diagnoses included cardiovascular causes such as acute coronary syndrome (ACS), aortic dissection, and pulmonary thrombosis. Laboratory results with troponin levels and D-dimers within the normal range, together with no pathological electrocardiogram changes, allowed the physicians to exclude the aforementioned diagnoses. Suspicion of infectious causes such as pneumonia was reduced due to low inflammatory markers in blood work. Further investigation with a chest X-ray was performed. Although the results did not reveal pneumonia or pneumothorax, a different interesting finding was seen. A chest CT revealed a massive sliding hiatal hernia with the gastric fundus protruding into the thoracic cavity (Figures 1, 2).

**Figure 3:**
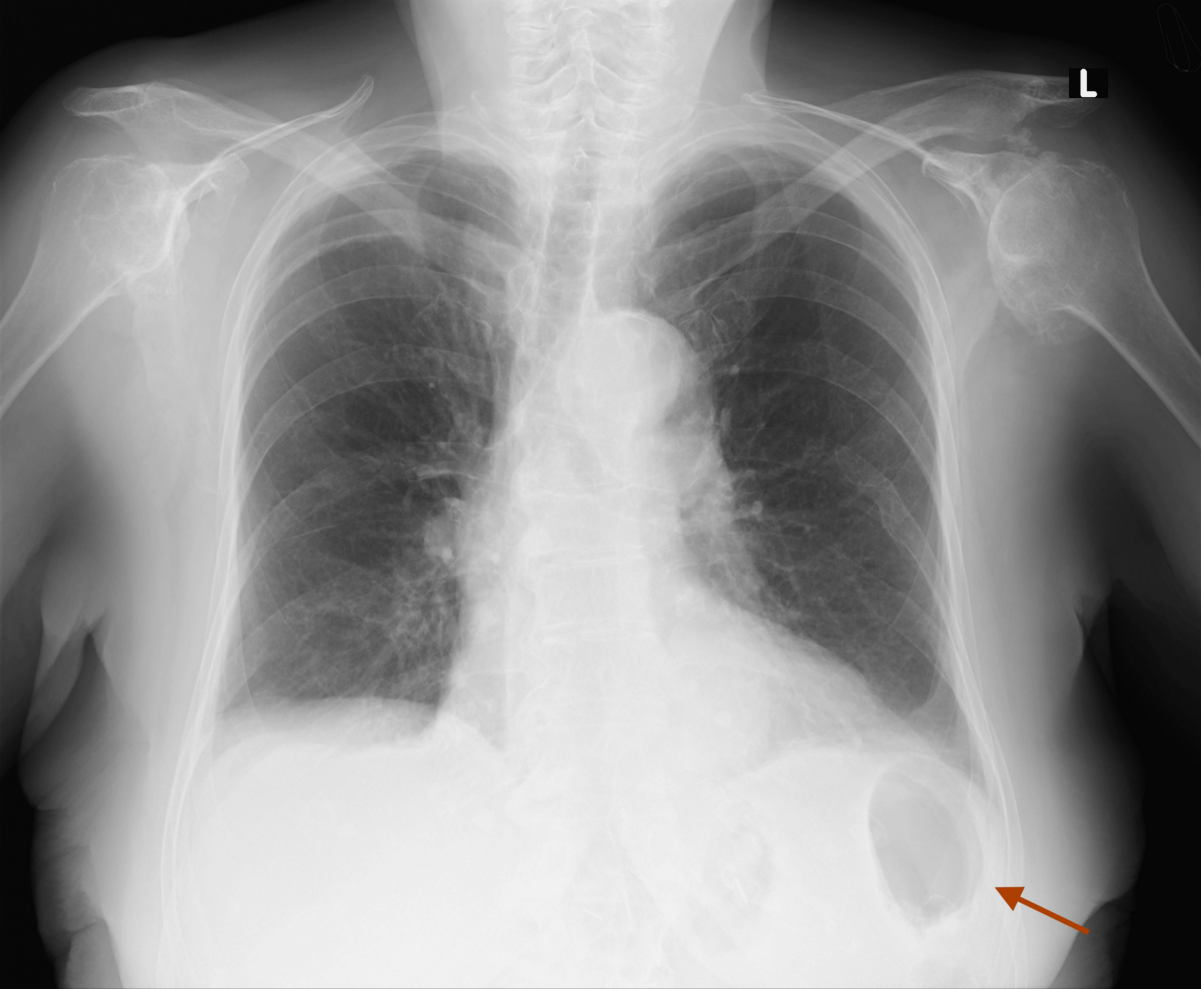
Follow-up chest X-ray with correct stomach position (gastric bubble).

The intrathoracic part of the stomach was distended with fluid, and the lumen of the esophagus was dilated as well. Moreover, the hernia caused mild cardiac compression and compression atelectasis of lower lung fields. The condition was considered life-threatening, and the patient was immediately qualified for urgent surgical repair. Based on her comorbidities, she was assessed as American Society of Anesthesiologists (ASA) class II. A laparotomy was performed, revealing the incarcerated hiatal hernia. The rim of the esophageal hiatus was incised to allow the stomach to be repositioned from the thoracic cavity into the abdominal cavity. The incarcerated part of the fundus was viable, with no signs of necrosis. The hiatal crura were approximated using single sutures, followed by a Nissen fundoplication, a procedure to prevent gastroesophageal reflux disease (GERD). In this technique, the surgeon wraps the fundus of the stomach around the lower esophagus, reinforcing the lower esophageal sphincter to reduce acid reflux. Additionally, the Nissen fundoplication helps prevent recurrent herniation of the stomach into the chest cavity. Laparoscopic treatment of incarcerated hiatal hernias can be more challenging and risky, especially in elderly patients, due to potential difficulties in safely dividing the esophageal hiatus without injuring the incarcerated stomach. There were no intraoperative or postoperative complications. The patient was transferred to the intensive care unit and remained intubated for one day. Oral feeding resumed on the second postoperative day, along with rehabilitation. The patient was discharged home in stable condition. After a four-month follow-up, the X-ray showed the correct position of the stomach in the abdomen, and the patient remains asymptomatic (Figure 3).

## Discussion

This case of a 95-year-old female cautions against a rare but possibly disastrous phenomenon, an incarcerated sliding hiatal hernia. Such a disease entity should be kept in mind among the differential diagnoses of chest pain. Furthermore, accurately recognizing a medical emergency in the elderly could still lead to a very successful surgical repair.

When an older patient reports chest pain and dyspnea, most physicians would correctly assume the symptoms could have a cardiovascular origin. While such a habit is extremely important to rule out acute, life-threatening diseases, it may be crucial to identify other possible underlying causes when all diagnostic examinations suggest otherwise. When the regular cardiac tests are negative, usually, a chest X-ray should follow. That is when a suspicion of an incarcerated hernia within the diaphragm could be drawn. A CT confirms the diagnosis. The same diagnostic route was taken with regard to our patient.

What could have preliminarily indicated the correct diagnosis in the present case was the anamnesis. The patient, a 95-year-old female with class I obesity, had various symptoms and risk factors indicative of a hiatal hernia. In the case of strangulation with compression of the neighboring organs, such an ailment may manifest with acute chest or epigastric pain and dyspnea, which agrees with the present case. The patient reported having had general fatigue for quite some time before admission, which could negate ACS. Furthermore, taking dysphagia into account could further lead us to gastroenterological causes. Moreover, it has been reported that the occurrence of a sliding hiatal hernia increases with age [4], is more prevalent among females, and may progress more often among those with higher body mass index [1]. A factor indicative of a possible hiatal hernia incarceration could also have been a history of previous surgeries, mainly gastrectomy (oncological and bariatric), which our patient had not undergone [6-10]. A fairly normal physical examination does not exclude incarceration since peritonitis is rarely seen due to anatomical positioning [11]. The signs of inflammation with fever, high C-reactive protein, and high white blood cell count would develop when the incarceration progresses to necrosis, therefore, the lack thereof does not change the preliminary diagnosis. However, since one always has to rule out cardiological emergencies, which share similar risk factors and symptoms, the diagnostic process should always begin with a basic physical examination, ECG, and laboratory workup, as it was done in the present case.

The literature states that among patients undergoing esophagogastroduodenoscopy, the number of hiatal hernias may amount to almost 30% [12]. However, the exact epidemiology for such hernia incarcerations is lacking. Most available data pertain to paraesophageal hernias and indicate that the annual risk of acute presentation in previously asymptomatic hernias is approximately 1% [5,13]. The suggested management of asymptomatic sliding hiatal hernias is watchful waiting [2]. However, in case of incarceration with tissue entrapment and possible ischemia may sometimes lead to disastrous consequences such as esophageal or gastric perforation [11,14] with subsequent septic complications [5,15]. A total fundoplication has been reported to bring the best results for a planned treatment of symptomatic hiatal hernia [3]. Nonetheless, information regarding the best course of management in incarcerated sliding hiatal hernias is scarce. In this case, the choice of a laparotomy was made based on the patient’s safety. Such an approach was considered the best technique for achieving the highest level of control over the procedure, given the patient’s high risk and the potential for intraoperative or postoperative complications.

## Conclusions

This study reports a successful, urgent surgical repair via laparotomy of a symptomatic, incarcerated sliding hiatal hernia in a 95-year-old female patient. It proves that sliding hiatal hernia incarceration requires further studies, as the symptoms, management, and prognosis may differ from what we already know regarding paraesophageal hernia. This case emphasizes the need for increased awareness among clinicians regarding the potential severity of sliding hiatal hernias. Although very rare, as the population is aging and becoming more obese, such emergencies may become more frequent, with those two aspects being key risk factors for such hernias. Additionally, this report suggests that surgical intervention, even in elderly patients, can be performed safely and effectively, leading to complete recovery.
